# Effect of Motor Interference Therapy on Distress Related to Traumatic Memories: A Randomized, Double‐Blind, Controlled Feasibility Trial

**DOI:** 10.1002/brb3.70063

**Published:** 2024-09-24

**Authors:** Alonso Morales‐Rivero, Daniel Crail‐Meléndez, Lorena Reyes‐Santos, Erik Bisanz, Jeffrey Bisanz, Angel Ruiz‐Chow, Monica Maritza Chavarria‐Medina

**Affiliations:** ^1^ Neurpsychiatric Department National Institute of Neurology and Neurosurgery Manuel Velasco Suarez Mexico City Mexico; ^2^ Centro Médico ABC Mexico City Mexico; ^3^ Faculty of Medicine Universidad Nacional Autonoma de Mexico (UNAM) Mexico City Mexico; ^4^ University of British Columbia Vancouver British Columbia Canada; ^5^ University of Alberta Edmonton Alberta Canada; ^6^ Hospital Star Médica Mérida Merida Yucatán Mexico

**Keywords:** motor interference therapy, non‐pharmacological treatment, posttraumatic stress disorder, traumatic memories

## Abstract

**Introduction:**

Traumatic memories (TM) are a core feature of stress‐related disorders, including posttraumatic stress disorder (PTSD). Treatment is often difficult, and specific pharmacological interventions are lacking. We present a novel non‐pharmacological intervention called motor interference therapy (MIT) as a promising alternative for these symptoms.

**Aims:**

To determine the feasibility of MIT, a brief, audio‐delivered, and non‐pharmacological intervention that uses cognitive and motor tasks to treat TM.

**Methods:**

We designed a randomized, double‐blind trial. Twenty‐eight participants from an outpatient clinic with at least one TM were included to receive either MIT or progressive muscle relaxation (PMR). Spanish versions of the PTSD symptom severity scale (EGS), visual analog scale for TM (TM–VAS), and quality of life (EQ–VAS) were applied prior to intervention, 1 week, and 1 month following intervention.

**Results:**

Mean scores on all measures improved from baseline to posttest for both groups. MIT participants showed significantly more positive scores at 1 week and 1 month (TM–VAS baseline: 9.8 ± 0.4; immediate: 6.0 ± 2.0; 1 week: 3.8 ± 3.1 [*d* = 1.57]; 1 month 2.9 ± 2.8 [*d* = 1.93]) than PMR participants on measures of distress due to TM, trauma re‐experiencing, anxiety, and a composite measure of PTSD.

**Conclusion:**

MIT is a simple, effective, and easy‐to‐use tool for treating TM and other stress‐related symptoms. It requires relatively few resources and could be adapted to many contexts. The results provide proof‐of‐principle support for conducting future research with larger cohorts and controls to improve clinical effectiveness and research on brief interventions.

**Trial Registration:**

ClinicalTrials.gov Identifier: NCT03627078

## Graphical abstract

Decrease in perceived distress when recalling the traumatic memory (TM), measured through the visual analog scale for TM (TM‐VAS). Participants who received motor interference therapy (red line) had a greater reduction in the distress generated by recalling the traumatic memory compared to the group that received progressive muscle relaxation (blue line).

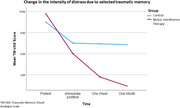

## 1 Introduction

Traumatic memories (TMs) are a common symptom found in stress‐related disorders, such as posttraumatic stress disorder (PTSD) and acute stress disorder (Brewin et al. 2010). They can be defined as recurrent, involuntary, and intrusive distressing memories of the traumatic event (American Psychiatric Association 2022). Subclinical levels of PTSD symptoms have many of the same negative outcomes as clinical levels, whereas treatment outcomes tend to be better in subclinical populations (Korte et al. 2016).

Treatment of PTSD symptoms poses many difficulties, including a shortage of practitioners, mental health resources, and aversion to seeking help due to fear of re‐experiencing trauma, as well as high levels of dropout and non‐response to interventions (Kazlauskas 2017). Although there are promising potential pharmacological treatments for symptoms of trauma, so far many approaches have failed to produce results, and evidence on the effectiveness of newer therapies is still lacking (Abdallah et al. 2019; Astill Wright et al. 2019).

One potential non‐pharmacological treatment of PTSD symptoms is the use of motor, cognitive, or visuospatial tasks to modify memory reconsolidation (Holmes et al. 2009; Holmes and Bourne 2008). As memories are recalled and reactivated, a reconsolidation process occurs that might be susceptible to modification (Sara 2000) using pharmacological or behavioral strategies (Walsh et al. 2018). In the last decade, various approaches have been taken to develop interventions that influence the reconsolidation process (Asselbergs et al. 2018; Horsch et al. 2017; Iyadurai et al. 2018).

Motor interference therapy (MIT) is a brief procedure that consists of a patient mentally retaining a negative or distressing thought or memory while being guided by an audio track directing them to complete simple motor and cognitive tasks. In a previous pilot study, 10 patients from a clinical population with TM showed statistically and clinically significant improvement 1 week after a single session of the intervention (Morales‐Rivero et al. 2020). To further evaluate the feasibility and effectiveness of the intervention, we conducted a double‐blind controlled trial of MIT with a comparison using a modified version of a common and well‐studied therapeutic technique, Jacobson's progressive muscle relaxation (PMR).

## 2 Methods

This study was conducted in accordance with the Helsinki Declaration of 1975, revised in 2008, and was approved by the Research Committee and Ethics Committee of the National Institute of Neurology and Neurosurgery of Mexico. The study's clinical trial registration number is NCT03627078 with ClinicalTrials.gov. Participant registration took place from March 2016 to March 2019. All participants provided written and informed consent to participate in this study.

Participants were recruited by referral from the general psychiatry outpatient clinic at the National Institute of Neurology and Neurosurgery in Mexico City. Patients were referred to by physicians who were informed of the study through posters and flyers. On the basis of the effect size of a previous study using MIT (1.42) *α* = 0.05 and 1 − *β* = 0.80, a sample size of 14 participants per group was required for the present study (Morales‐Rivero et al. 2020). Inclusion criteria were age (at least 16 years old, as this is the minimum age required to receive medical care at the Institute, which is the minimum age for the outpatient program), Spanish as a first language, and at least one TM that caused distress. We defined TM as recurrent, involuntary, and intrusive distressing memories of a traumatic event (American Psychiatric Association 2022, 0 5).

People who were under psychopharmacological treatment could participate if they had not started or had any change in their medication in the last 8 weeks. Patients with any neurological or psychiatric disorder impacting verbal comprehension or judgment, or with any hearing impairment were excluded.

## 3 Procedures

### 3.1 Participants and Measures

At the first visit, a researcher conducted an initial interview, explained the study, and the patient gave informed consent. If they were eligible for the study, a second visit was scheduled for the intervention, and all measurement scales were applied. Patients with several memories were instructed to select the one that bothered them the most. Participants were then randomly assigned to receive MIT or PMR using a Microsoft Excel randomization list. Both interventions consisted of 14‐min audio tracks. The audios were played twice for a total of 30 min, with a short pause in between. We used Bose noise‐canceling headphones and itunes from a MacBook pro to play both audios. The researcher monitored task completion and recorded the duration of the sessions. The participants in both conditions completed at least 80% of the task. A rater, blind to the intervention, administered all scales 1 week (third visit) and 1 month after the intervention (fourth visit). The TM–VAS was also administered immediately after the intervention. Participants were asked not to comment on the intervention to the evaluators. All interventions took place in the same room. Patients continued their care at the correspondent outpatient clinic from which they were referred to.

### 3.2 Motor Interference Therapy

The MIT group listened to a 14‐min audio track. The first 4 min of this track instructed the subjects to tap their fingers on a table in response to specific audio cues: right‐handed tapping with high‐pitched fast sounds, left‐handed tapping with high‐pitched slow sounds, and bilateral tapping with the low‐pitched continuous tone, while recalling a specific TM. During the remaining 10 min, patients were asked to perform a series of motor tasks involving both hands, as the finger tapping was guided by specific sounds and directions that were presented in a random order, whereas a female voice instructed them to recall the memory and perform the tapping at the same time. The audio was applied twice in the same session. (Morales‐Rivero et al. 2020).

### 3.3 Progressive Muscle Relaxation

Participants in the other group underwent a modified short version of Jacobson's PMR technique, which consists of two steps. The first step is to intentionally tense specific muscles to understand the sensation of tension in those areas. The next step is to release the tension while observing the sensation of relaxation as it occurs. By systematically tensing and relaxing different muscle groups in sequence, individuals learn to identify and distinguish between the sensations of tensed and relaxed muscles (Jacobson 1925). We used an audio of the same length as the MIT. The first 4 min were instructions on how to perform the PMR exercises. For the next 10 min, participants were asked to recall a TM while engaging in PMR, also guided by a female voice.

## 4 Measures

Scales were selected to assess both PTSD‐related symptoms and patients’ perceived quality of life. The scales used for PTSD symptoms were from the Spanish version of the PTSD symptom severity scale (EGS: *Escala de Gravedad de Síntomas*) (Odriozola et al. 1997). The EGS consists of 17 questions arranged in 3 subscales that reflect PTSD criteria. Subscale scores are summed to yield a composite score of 0–51, with a total of 15 or greater indicating a probable diagnosis of PTSD. The subscales are Re‐experiencing (e.g., “Do you have unpleasant and recurrent memories of the event, including images, thoughts or perceptions?”), Avoidance (e.g., “Do you make efforts to avoid thoughts, feelings, or conversations associated with the event?”), and Arousal (e.g., “Do you startle or become alarmed more easily since the event?”). The EGS also includes a supplementary scale assessing anxiety symptoms with a possible score of 0–39. This instrument demonstrates strong concurrent and divergent validity, construct validity, and consistency, with a Cronbach's alpha of 0.92 and a test–retest reliability at 4 weeks of 0.89. It has often been used in both therapeutic and research contexts.

To measure health‐related quality of life, we used the visual analog scale (EQ–VAS) from the EuroQol‐5D (The Euroqol Group 1990), which asks a patient to rate their overall health on that day on a thermometer‐like scale from 0 to 100, where 0 is the worst health imaginable, and 100 is the best health imaginable. This part of the EuroQol‐5D is considered a valid measure of health from the patient's perspective (Feng, Parkin, and Devlin 2014).

Participants rated the intensity of distress generated by their selected TM with the (TM–VAS, a simple visual‐analog scale from 0 (no unpleasant feeling at all) to 10 (most unpleasant feeling possible), presented in a similar way as EQ–VAS, which was used in a previous study (Morales‐Rivero et al. 2020).

## 5 Statistical analyses

For each dependent variable (EGS, EQ–VAS, and TIM–VAS scores) a Group (PMR, MIT) × Session analysis of variance was conducted with repeated measures on the second variable. Critical interactions of Group and Session were further analyzed with pairwise least significant differences tests. Analyses of covariance were conducted to evaluate posttest scores adjusted for baseline differences, presuming that baseline differences were simply due to measurement error. Tests of sphericity were conducted for all ANOVAs and all but one were nonsignificant. In the case of one significant test, standard corrections for non‐sphericity did not change the pattern of significant outcome.

Effect sizes (Cohen's *d*) were computed separately for the PMR and MIT interventions by comparing differences from baseline to 1 week and from baseline to 1 month, which provides insight if group differences at baseline were true despite randomization. SPSS Version 28 for windows was used for all analyses (IBM Corp. Released 2021. IBM SPSS Statistics for Windows, Version 28.0. Armonk, NY: IBM Corp n.d.).

## 6 Results

Thirty‐four patients were evaluated for eligibility. Three were excluded because they did not meet the inclusion criteria; one withdrew informed consent before the intervention, and two others chose not to participate. None withdrew once the study was underway. A flow chart is included in the Supporting Information section.

The sample population consisted of 27 females and 1 male ranging in age from 18 to 62 years (Table 1). The majority of the patients had psychiatric comorbidities made by the referring physician. Socioeconomical status is determined by Department of Social Work of the National Institute of Neurology and Neurosurgery.

**TABLE 1:** Demographic and clinical characteristics.

	MIT (*n* = 14)		PMR (*n* = 14)	
Variable	Mean (SD)	%	Mean (SD)	%
Age	42.3 (14.2)		35.4 (14.5)	
Years of schooling	13.4 (4.7)		13.3 (5.1)	
Low socioeconomic status		71		79
Marital status: single		64		79
Psychiatric comorbidities		86		86
PTSD diagnosis		79		71
Personality disorder		14		14
Any anxiety disorder		14		21
Major depressive disorder		57		50
Pharmacological treatment		79		79
Any previous psychotherapy		36		50

Abbreviations: MIT, motor interference therapy; PMR, progressive muscle relaxation; PTSD, posttraumatic stress disorder.

All participants but one were in the critical range for PTSD, based on the assessment tool used in the study. Psychiatric diagnoses other than PTSD were obtained from medical records. No significant differences were found in 14 demographic and clinical characteristics between the two groups, as shown in Table 1 (demographics and clinical variables) and Table 2 (measures).

**TABLE 2:** Means, standard deviations, and effect sizes across sessions.

Measure		Baseline		Immediate		1 Week			1 Month		
	Group	Mean	SD	Mean	SD	Mean	SD	*d* (CI)^a^	Mean	SD	*d* (CI)^a^
TM–VAS	MIT	9.8	0.4	6.0	2.0	3.8	3.1	1.57	2.9	2.8	1.93
	PMR	9.0	0.9	7.0	2.4	6.9	2.0	(0.70–2.41)	6.9	2.1	(1.01–2.82)
EGS Composite	MIT	40.1	5.4			21.4	11.2	1.23	18.4	9.9	1.43
	PMR	30.6	10.4			23.7	10.8	(0.41–2.03)	22.8	11.0	(0.59–2.26)
EGS Arousal	MIT	12.8	2.2			7.8	4.1	0.96	7.4	4.0	0.96
	PMR	9.6	2.7			7.9	3.3	(0.16–1.73)	7.7	2.9	(0.16–1.73)
EGS Avoidance	MIT	16.2	3.5			8.8	5.6	0.87	7.5	4.6	1.08
	PMR	11.8	6.1			8.7	5.9	(0.09–1.64)	8.8	5.5	(0.27–1.87)
EGS Re‐Experiencing	MIT	11.1	2.5			4.8	3.0	1.32	3.6	2.8	1.58
	PMR	9.0	3.7			6.9	3.7	(0.49–2.13)	6.3	3.5	(0.71–2.42)
EGS Anxiety	MIT	21.9	8.4			13.8	11.0	.74	9.9	8.4	1.23
	PMR	19.6	10.8			16.4	10.5	(0.04–1.50)	16.0	10.0	(0.41−2.03)
EQ‐VAS	MIT	43.9	21.9			69.3	17.2	0.51	69.4	24.4	.48
	PMR	44.7	34.6			60.5	26.0	(−0.24–1.26)	58.2	20.7	(−0.27–1.23)

*Note*: For the TM‐VAS and the EQ‐VAS, higher scores denote more positive outcomes. For the EGS scales, lower scores denote more positive outcomes.

Abbreviations: EGS, Spanish version of the posttraumatic stress disorder symptom severity scale; EQ–VAS, visual analog scale for quality of life; MIT, motor interference therapy; PMR, progressive muscle relaxation; TM–VAS, visual analog scale for traumatic memories.

^a^ Effect sizes (Cohen's *d* and 95% confidence interval) for the difference between improvement in the MIT group and improvement in the PMR group.

### 6.1 Effect on the Distress Generated by Traumatic Memories

**FIGURE 1:**
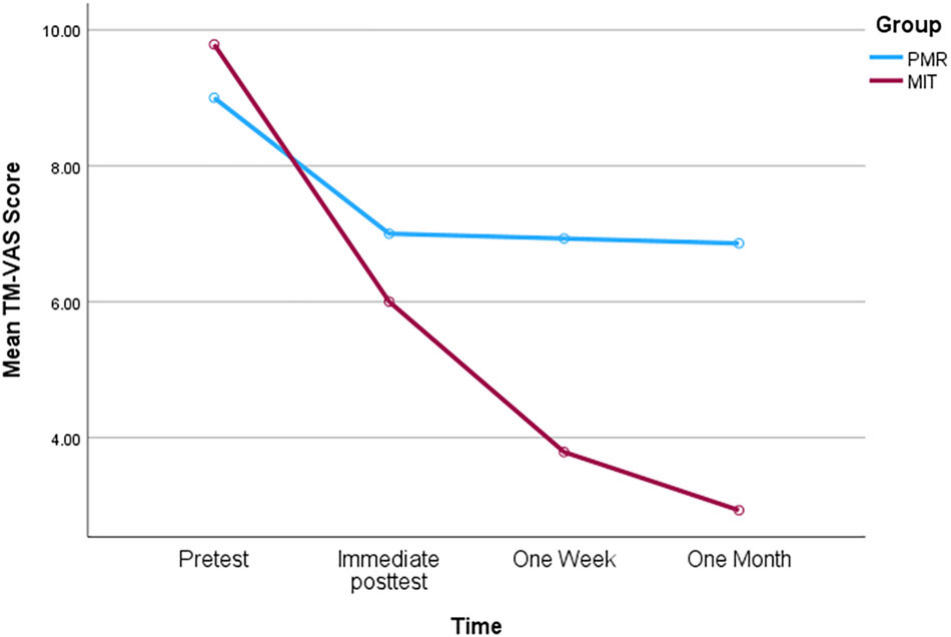
Mean TM–VAS scores as a function of group and session. TM–VAS, visual analog scale for traumatic memories.

For the TM–VAS, lower scores reflect less distress related to the TM (Figure 1). Mean scores decreased across sessions, *F*(3,78) = 42.41, *p* < 0.001, *η*_p_^2^ = 0.62, PMR scores were, on average, higher than MIT scores, *F*(1,26) = 8.95, *p* = 0.006, *η*_p_^2^ = 0.26, and Group and Session interacted, *F*(3,78) = 11.81, *p* < 0.001, *η*_p_^2^ = 0.31. Means for the two groups differed at baseline, 1‐week posttest, and 1‐month posttest (*p*s < 0.01). PMR scores declined from baseline to the immediate posttest (*p* = 0.002) but showed no further decline. MIT scores also declined from baseline to immediate posttest (*p* < 0.001), but they continued to improve over the subsequent week (*p* = 0.002) and were sustained 1 month following treatment (*p *= 0.001) The subsequent analysis of covariance, with baseline score as the covariate, revealed that MIT scores across posttests were lower than PMR scores, *F*(1,25) = 15.35, *p* < 0.001, *η*_p_^2^ = 0.38, and that this effect varied across the three posttest sessions, *F*(2,50) = 3.86, *p* = 0.028, *η*_p_^2^ = 0.13. Specific comparisons confirmed that MIT scores decreased from immediate posttest to the 1‐week posttest (*p* = 0.004) and remained stable thereafter, PMR scores were constant across all three posttests, and mean MIT scores were lower than mean PMR scores at 1 week and 1 month (*p*s < 0.002).

### 6.2 Effect on PTSD Symptoms

For all EGS measures, lower scores reflect more positive outcomes. Composite scores on the EGS symptom severity scale decreased across sessions, *F*(2,52) = 45.67, *p* < 0.001, *η*_p_^2 ^= 0.64, overall means for the two groups did not differ, *F*(1,26) < 1, and Session interacted with Group, *F*(2,52) = 9.91, *p* < 0.001, *η*_p_^2 ^= 0.28. PMR and MIT scores both decreased from baseline to the 1‐week posttest (*p*s < 0.02) and were stable thereafter, but the drop from the baseline to 1 week posttest was greater for the MIT group. MIT scores were higher than PMR scores only at baseline (*p* = 0.005). When posttest scores were adjusted for this difference, however, the MIT group outperformed the PMR group on both posttests combined, *F*(1,25) = 6.32, *p* = 0.019, *η*_p_^2 ^= 0.20.

Scores on the EGS subscale for arousal decreased across sessions, *F*(2,52) = 18.90, *p* < 0.001, *η*_p_^2^ = 0.42, the difference between overall means for the two groups was negligible, and Session and Group interacted, *F*(2,52) = 4.66, *p* = 0.014, *η*_p_^2^ = 0.15. Scores decreased from the baseline to posttests for both groups, but the decline was reliable only for the MIT group (*p* < 0.001). The two groups differed reliably only at baseline (*p* = 0.002). When posttest scores were adjusted for baseline differences, however, mean MIT scores were lower than mean PMR scores, but again the differences were not reliable.

Scores on the EGS subscale for avoidance decreased over time, *F*(2,52) = 25.94, *p* < 0.001, *η*_p_^2 ^= 0.50, overall group means did not differ reliably, and the two variables interacted, *F*(2,52) = 5.57, *p* = 0.006, *η*_p_^2 ^= 0.18. Scores decreased from baseline to 1‐week posttest for both PMR (*p* = 0.03) and MIT (*p* < 0.001) and were sustained at 1 month, but the decrease from first to second test was much greater for the MIT group. The difference between groups was reliable only at baseline (*p* = 0.026). When posttest scores were adjusted for baseline differences, the lack of group differences on both posttests was confirmed.

Scores on the EGS subscale for re‐experiencing decreased over time, *F*(2,52) = 53.09, *p* < 0.001, *η*_p_^2^ = 0.67. Overall group means did not differ, but Group and Time interacted, *F*(2,52) = 12.45, *p* < 0.001, *η*_p_^2^ = 0.32. PMR scores declined from baseline to the 1‐week test (*p* = 0.025) and remained stable to 1 month. MIT scores decreased more precipitously from baseline to the 1‐week test (*p* < 0.001) and again to the 1‐month posttest (*p* = 0.027). Means for the two groups differed only at the 1‐month posttest (*p* = 0.032). When posttest scores were adjusted for baseline differences, means for the two groups differed for both posttests combined, *F*(1,25) = 11.11, *p* = 0.003, *η*_p_^2^ = 0.31.

Scores on the EGS scale for anxiety decreased over time across testing intervals, *F*(2,52) = 22.46, *p* < 0.001, *η*_p_^2^ = 0.46, means for the two groups differed negligibly, and Session interacted with Group, *F*(2,52) = 6.04, *p* = 0.004, *η*_p_^2^ = 0.19. MIT scores declined from baseline to 1‐week posttest (*p* < 0.001) and again to 1‐month posttest (*p* = 0.017). Changes over time were not reliable for the PMR group, and neither were group differences at any of the time intervals. When posttest scores were adjusted for the small differences at baseline, mean MIT scores were lower than mean PMR scores on both delayed posttests combined, *F*(1,25) = 7.71, *p* = 0.01, *η*_p_^2^ = 0.24.

### 6.3 Effect on Quality‐of‐Life Judgments

For the quality‐of‐life measure, higher scores reflect more positive outcomes. Mean EQ–VAS scores increased across testing periods, *F*(2,52) = 8.71, *p* < 0.001, *η*_p_^2^ = 0.251, but the main effect of group and the interaction were negligible. The improvement for both groups at 1 week was maintained at 1 month. Group differences were negligible at all times of testing. The subsequent analysis adjusting for baseline scores confirmed that group differences at 1 week and 1 month were unreliable.

## 7 Discussion

In this study, we sought to evaluate the feasibility of MIT, a simple and seemingly well‐tolerated novel interventions as compared with a conventional treatment. Mean scores improved from baseline to posttests for both interventions on all measures. These improvements were statistically significant in all seven cases for MIT and in five of seven cases (not Arousal and Avoidance) for PMR. In every case where an improvement was confirmed, it was maintained 1 month post intervention.

Mean scores for the MIT group were better than those for the PMR group after 1 week and 1 month, and for four of seven measures (TM–VAS for TM, EGS Composite, Re‐Experiencing, and Anxiety), these differences were reliable even after adjusting for baseline differences. Effect sizes comparing improvements in the MIT group with those in the PMR group were substantial for most measures. The advantage of MIT over PMR was most pronounced for the measure of distress caused by TM, for the composite measure of PTSD symptoms, and for the scales reflecting anxiety and re‐experiencing traumatic events.

PMR has been used as a comparison in interventional studies for stress‐related disorders (Carletto et al. 2016; Conrad and Roth 2007; Ranney et al. 2022). The fact that both interventions showed improvement could be related to several factors, such as patient expectations, novelty of the treatment, therapeutic relationship, or even motivational and placebo effects inherent in any treatment. (Finniss et al. 2010; Mulder et al. 2017); however, the MIT intervention showed better outcomes.

In this study, factors such as the length of the intervention, blinded raters, and the same time frame for evaluating patients were controlled to make both conditions as similar as possible. Again, this suggests that the posttest differences between the two groups were due to the effect of the MIT and not just the personalized attention and improvement over the usual standard of care (Vanderpool 2021). In the present case, participants with persistent symptoms volunteered for a trial of a new treatment approach and received attention from clinicians who asked about their experiences and administered clinical scales, all of which could influence engagement and expectancy (Wampold 2007). This new alliance may also explain the improvements in both interventions (Cuijpers, Reijnders, and Huibers 2019).

The greater effect of the MIT group could be due to the motor‐cognitive task competing for resources required not only to reconsolidate memories, but also to the interference of intrusive mechanisms of the memory helping to reduce distress (Deeprose et al. 2012; Iyadurai et al. 2018; Kessler et al. 2020). The verbal instructions on the TIM track include specific suggestions that the TM is changing, which may have influenced the remission of symptoms adding to the effect of imaginal exposure, which itself is emerging as an effective brief intervention for PTSD symptoms (Zoellner et al. 2023).

Other interventions, such as eye movement desensitization and reprocessing therapy (EMDR), depend on the experience of the therapist (Chen et al. 2014), whereas no prior experience is required in our application. Other studies of PMR and EMDR have shown improvement in stress‐related symptoms, but at least 10 sessions were required (Carletto et al. 2016) in contrast to our 30‐min intervention.

### 7.1 Limitations

The sample sizes were sufficient to identify important differences between groups but perhaps not large enough to enable the detection of differences that were suggestive but not statistically significant. The groups also had unfortunate baseline differences, and the exact effect cannot be determined due to the lack of a non‐treatment control. Even so, the results of this study are consistent with the previous pilot study and come in the context of strong results in emerging areas of study such as brief interventions using analogous techniques that likely alter memory reconsolidation (e.g., Iyadurai et al. 2020; Kessler et al. 2020; Ramineni et al. 2023; Van Bentum et al. 2019), and brief or single‐session interventions in mental health (e.g., Schleider et al. 2021; Schleider, Smith, and Ahuvia 2023; Zoellner et al. 2023).

The sample is heavily skewed toward women, which is somewhat consistent with a higher proportion of women with symptoms of trauma (Christiansen and Berke 2020) and the prevalence of intrusive memories related to trauma in women (Iyadurai et al. 2019), and a lower proportion of men seeking treatment for mental health problems (Bilsker et al. 2018; Güney et al. 2024; Nam et al. 2010). Beyond that, we do not have a more complete explanation for why such a disproportionate number of women were referred to the study.

### 7.2 Future Implications

The results of this study are consistent with the previous pilot study (Morales‐Rivero et al. 2020) and come in the context of strong results in emerging areas of study such as brief interventions using analogous techniques that likely alter memory reconsolidation (Iyadurai et al. 2020; Kessler et al. 2020; Ramineni et al. 2023; Van Bentum et al. 2019), and brief or single‐session interventions in mental health (Schleider et al. 2021; Schleider et al. 2023; Zoellner et al. 2023). Promising recent developments in psychological science—including self‐applied and scalable technologies—have not made their way into psychological treatments in spite of an acute need for them (Holmes et al. 2018).

Future studies could be designed to determine whether stacking different known elements would produce an enhanced effect in a short period of time. Brief interventions with promising potential are psychoceuticals (Cano et al. 2022) and their combination with specific treatment modalities (Lucke‐Wold et al. 2018). It may be interesting to consider the combination of MIT with the pharmacological effects of psychoceuticals as a possible enhancer of the effect.

### 7.3 Clinical Implications

There has long been a global shortage of mental health care practitioners (Weil 2015), a situation exacerbated by the COVID‐19 pandemic (Galea, Merchant, and Lurie 2020). Although there are promising potential pharmacological treatments for symptoms of trauma, so far many approaches have failed to produce results, and evidence on the effectiveness of newer therapies is still lacking (Abdallah et al. 2019; Astill Wright et al. 2019; Cano et al. 2022). MIT could be a reliable option in fulfilling this unmet need.

Interventions that interfere with memory reconsolidation, such as MIT, seem safe and effective for self‐applied use (Kessler et al. 2018), and digital applications are already being developed for self‐application (Asselbergs et al. 2018; Ramineni et al. 2023).

MIT has features that may make it especially well‐suited for bridging the gap between research findings and clinical applications as it is brief, can be clinician‐ or self‐applied, and blindable. As a self‐delivered audio intervention, future component analyses could effectively study both specific and nonspecific effects of the intervention, which is difficult or impossible with most non‐pharmacological interventions (Wampold and Imel 2015).

## 8 Conclusion

Our initial investigations show that MIT is an easy‐to‐apply technique that requires few resources and may be adaptable to many contexts. It compared favorably with a well‐known intervention and had no adverse effects, suggesting that it could be a relatively safe process with many potential benefits to both patients and health care practitioners. Further research with larger groups could help improve clinical effectiveness and add to a growing body of research around brief interventions.

## Author Contributions

**Crail‐Meléndez Daniel** conceived the presented idea and designed the implementation of the research, contributed with data analysis, supervised the findings of this work, drafted the manuscript, and designed the figures. **Morales‐Rivero Alonso** and **Reyes‐Santos Lorena** designed the implementation of the research, recruited the sample, performed the intervention collected the data, contributed with data analysis, drafted the manuscript, and help designing the figures. **Bisanz Erik** conceived the presented idea, designed the implementation of the research, and drafted the manuscript. **Jeffrey Bisanz** contributed performing and interpreting the data analysis as well as contributed to the drafting and critical review of the article**. Ruiz‐Chow Angel** designed the implementation of the research, performed the intervention, collected the data, and contributed with data analysis. **Chavarria‐Medina Monica Maritza** contributed with the implementation of the research, data analysis, and drafted and revised the document. All authors discussed the results and contributed to the final manuscript.

## Ethics Statement

This human study was performed in accordance with the Helsinki Declaration of 1975, as revised in 2008, and was approved by Research committee and ethics committee of the National Institute of Neurology and Neurosurgery.

## Consent

Participant registration took place from March1, 2016 to March 30, 2019. All adult participants provided written informed consent to participate in this study.

## Conflicts of Interest

Daniel Crail‐Meléndez, Alonso Morales‐Rivero, Lorena Reyes‐Santos, Jeffrey Bisanz, and Angel Ruiz‐Chow declare no conflicts of interest. Erik Bisanz has given advice on a voluntary basis to Orpheus Mind Technologies Limited, which currently holds copyright of the audio tracks used in Motor Interference Therapy.

### Peer Review

The peer review history for this article is available at https://publons.com/publon/10.1002/brb3.70063.

## Supporting information



Supporting Information

## Data Availability

The data that support the findings of this study are available from the corresponding author, DCM, upon reasonable request.
