# Supplementary material for: Effect of Motor Interference Therapy on Distress Related to Traumatic Memories: A Randomized, Double‐Blind, Controlled Feasibility Trial
Source: Brain Behav. 2024 Sep 24;14(9):e70063. doi: 10.1002/brb3.70063 (PMC11422178; doi:10.1002/brb3.70063)
Supplement: Supplementary file 1 — Supporting Information [file BRB3-14-e70063-s001.docx]

**CONSORT Flow Diagram**

## Follow-Up

Analysed (n= 14 )
♦ Excluded from analysis (give reasons) (n=0 )

## Analysis

Analysed (n=14 )
♦ Excluded from analysis (give reasons) (n=0 )

Lost to follow-up (give reasons) (n= 0 )

Discontinued intervention (give reasons) (n=0 )

Lost to follow-up (give reasons) (n= 0 )

Discontinued intervention (give reasons) (n=0 )

## Enrollment

Allocated to intervention (n=14 )

♦ Received allocated intervention (n=14 )

♦ Did not receive allocated intervention (give reasons) (n=0 )

## Allocation

Allocated to intervention (n= 14 )

♦ Received allocated intervention (n=14 )

♦ Did not receive allocated intervention (give reasons) (n=0 )

Randomized (n=28 )

Excluded (n=6 )

♦  Not meeting inclusion criteria (n=3 )

♦  Declined to participate (n=1 )

♦  Other reasons (n=2 )

Assessed for eligibility (n=34 )
